# Benefits and issues of education program for nurse-midwives on milk expression care for preterm mothers in postpartum period

**DOI:** 10.1016/j.heliyon.2022.e11072

**Published:** 2022-10-15

**Authors:** Rie Tanaka, Shigeko Horiuchi

**Affiliations:** a Graduate Course of Midwifery, Teikyo University, Tokyo, Japan; b Graduate School of Nursing Science, St. Luke’s International University, Tokyo, Japan

**Keywords:** Preterm birth, Breast milk expression, Obstetric nursing, Knowledge, Education program

## Abstract

**Background and aims:**

Breastfeeding is important for preterm mothers and infants. However, evidence-based practice and standardized education remain inadequate. To implement evidence-based practice, continuous education is mandatory for nurse-midwives. We previously implemented our developed education program on early essential milk expression care for preterm mothers among Japanese nurse-midwives. Herein, we aimed *to assess* the effects of our education program on *nurse-midwives'knowledge of milk expression care for preterm mothers* in terms of changes in their correct answer rates for 20 specific knowledge items before and after the education program implementation. We also aimed *to identify* program-related issues from nurse-midwives' comments to improve the program.

**Methods:**

We conducted a secondary analysis of our previous data and surveyed the knowledge of 36 nurse-midwives who received a similar face-to-face education program on milk expression care for mothers three months before (Pre-1), just before (Pre-2), just after (Post-1), and three months after (Post-2) the program. We obtained their comments at Post-2 and identified issues for program improvement.

**Results:**

The knowledge items, in which the correct answer rates of Post-1 were significantly higher than those of Pre-2, were Verification of the motivation and intent, Benefits of breastfeeding for mothers and infants, Milk volume on the fourth day and at around the second week after birth, Lactogenesis stage 3, Autocrine control, Time between birth and the initiation of milk expression, Early initiation of milk expression, Frequent milk expression, and Duration of pumping. The issues identified were practical training, knowledge retention, and misunderstanding knowledge.

**Conclusions:**

Nurse-midwives' unacquired knowledge of milk expression care for preterm mothers was effectively supplemented by the education program. Pre-education knowledge items with low correct answer rates must be strengthened during in-service education. Practical training, knowledge retention, and misunderstanding knowledge can be improved.

## 1 Introduction

To translate research evidence into improvements in the health outcomes of patients, healthcare professionals must understand and apply the following process: (1) become aware of the best evidence; (2) accept the best evidence; (3) apply the evidence; (4) ensure the availability of the best evidence, and (5) act on the best evidence [1]. Therefore, continuous education for healthcare professionals is important to achieve evidence-based practice. However, insufficient, inconsistent, and non-standardized education on breastfeeding support has been reported [2, 3, 4]. Additionally, there has been a lack of evidence on the effectiveness of breastfeeding education for healthcare staff in supporting breastfeeding mothers [5,6]. The “ten steps to successful breastfeeding” of the World Health Organization and United Nations Children’s Fund [7] indicated that it is important that “staff have sufficient knowledge, competence and skills to support breastfeeding” (p. 8). Notably, continuing education on breastfeeding was found to be meaningful in improving the knowledge and clinical practice of nursing professionals [8]. This means that conducting an education program focusing on breastfeeding based on research evidence can promote evidence-based practice of nurses and nurse-midwives.

As previously identified, human milk has many benefits for infant growth such as development of cognitive function, decreased risk of overweight and obesity, and reduced risk of type 2 diabetes [9, 10]. In the case of preterm infants, human milk reduces the risk of necrotizing enterocolitis [11]. The American Academy of Pediatrics [12] recommended exclusive breastfeeding for about six months from the perspective of reduced risk of sepsis, necrotizing enterocolitis, severe retinopathy of prematurity, metabolic syndrome, long-term growth failure, and neurodevelopmental disorders. Moreover, the advantages of breastfeeding for mothers include lower risk for premenopausal breast cancer [13] and less metabolic syndrome [14]. Therefore, it is important to support breastfeeding for the health of preterm mothers and infants.

Depending on the gestational age of the infant, mothers have to express breast milk for their infants as most preterm infants are unable to latch on their mothers' nipples effectively and transfer milk from the breast. Therefore, for preterm mothers, guidance of breast milk expression is needed to initiate and maintain lactation. Preterm mothers already have the risk of producing less breast milk at the sixth week after birth compared with term mothers [15]. It was also reported that breast milk volume in the early puerperium period significantly predicted the subsequent breast milk volume [16, 17, 18]. Regarding the milk expression care to promote milk production, the following forms of care were proved to be effective: (a) starting milk expression within 1 h after delivery if possible [19, 20, 21], (b) securing milk expression seven or more times [19, 22, 23], (c) combining hand expression (more than five times a day) with electric pumping until the onset of lactogenesis 2 if using an electric pump [23]. These forms of care for mothers in the early postpartum period help maintain their proper amount of milk until their babies are sufficiently strong to latch on and suckle the breasts.

However, the early start of milk expression after birth has not been rigorously practiced in Japan to date [24, 25]. In a survey on breastfeeding support conducted among head nurses in Japanese neonatal intensive care units (NICUs), 77.6% of the nurses who gave guidance were obstetric nurses [26]. Unfortunately, a previous pilot study indicated that there are no clear standards of care to start and continue milk expression in a Japanese obstetric ward [25]. Obstetric nurse-midwives provided care for mothers without sufficient understanding of the situation of milk expression among mothers [25]. Nevertheless, nurse-midwives working in obstetric wards have to play an important role in initiating preterm mothers' milk expression. The appropriate nursing care for breast milk expression is mandatory to start milk expression as soon as possible and promote preterm mothers' milk production. Therefore, we previously designed and implemented an education program for nursing professionals on the importance of milk expression care for preterm mothers in the early puerperium period using a pre-post interventional study in one group. The implementation of the education program significantly enhanced the knowledge, attitude, and care implementation of nurse-midwives [27].

In the present study, we performed a secondary analysis of the data of our previous study [27], which involved the assessment of 20 knowledge items of the developed education program. Our specific aims were *to assess* the effects of our educational program on *nurse-midwives'* *knowledge of breast milk expression care for preterm mothers* in terms of changes in their correct answer rates for 20 specific knowledge items before and after implementation of the education program, and *to identify program-related issues* based on the nurse-midwives' comments that can be considered for improving the program.

## 2 Materials and methods

### 2.1 Study design

We performed an exploratory secondary analysis of the data obtained in our previous pre-post interventional study in one group [27]. In our previous study, the primary outcome was the difference in knowledge score between just before and just after the education program at a sample size of 34. In the present study, there are no primary outcomes or set sample size, being an exploratory study. This involves *assessment of* the effects of our education program on *nurse-midwives'* *knowledge of breast milk expression care for preterm mothers* in terms of changes in their correct answer rates for 20 specific knowledge items (Q1-Q20) before and after the program implementation. This also entails *identification of program-related issues* based on the nurse-midwives' comments for improving the program.

### 2.2 Inclusion and exclusion criteria

We recruited nurses and nurse-midwives providing care for preterm mothers in the obstetric ward of a perinatal medical center in a metropolitan area in Japan regardless of the years of experience or whether they have an International Board Certified Lactation Consultant (IBCLC) certification in the last part of June to July 2018. Although the years of experience and IBCLC certification were considered to be factors affecting knowledge level, changes in individual knowledge level brought about by the education program are anticipated to occur. Therefore, we have determined that if nurses and nurse-midwives with many years of experience or IBCLC certification were willing to participate in an education program based on the explanations of the study, they did not need to be excluded. In the obstetric ward of the research collaboration facility, about 50 nursing professionals were working, and more than 1000 births per year occurred.

### 2.3 Education program

We previously developed an education program for nursing professionals on appropriate milk expression care for preterm mothers on the basis of the Japanese guidelines [28] and previous studies [16, 17, 19, 20, 21, 22, 23, 29]. The education program included nursing care which should be implemented mainly in the obstetric ward after birth for preterm mothers. We indicated the contents of the education program in Box 1 [27]. Lecture materials were created as slides and distributed to the participants. The slides consisted of text, figures, and tables. The lead researcher (RT) provided face-to-face education for 60 min from the middle part of October 2018 to the last part of November 2018. All the participants attended one similar face-to-face education session.

Box 1 **Contents of the education program**

A) Emotional support
  Understanding the mother’s mental state
  Receptive, empathetic, and considerate attitude
B) Respect for mothers' decision-making
  Confirming the mother’s willingness
  Increasing the mother’s motivation
C) Understanding the characteristics of breast milk and significance of breastfeeding
  Benefits of breastfeeding
  Characteristics of breast milk among mothers of preterm infants
D) Provision of information related to the necessity and methods of milk expression and assistance in the implementation
  Significance of milk expression for mothers
  Importance of milk production in the early postpartum period
  Mechanism of milk production and secretion
  Early milk expression: within one hour after birth
  Frequent milk expression: seven or more times per day
  Duration of pumping: exceeds 100 minutes per day
  Addition of hand expression: more than five times a day during lactogenesis 1 if using an electric pump
  Selection of a comfortable method for mothers
  Methods of hand expression
E) Mental support and provision of information to mothers who cannot breastfeed
F) Introduction of social resources related to breastfeeding

(adapted from Tanaka & Horiuchi, 2021)

Alt-text: Box 1

### 2.4 Data collection

Three months before (**Pre-1**; last part of June 2018 to middle part of August 2018), just before (**Pre-2**; middle part of October 2018 to last part of November 2018), just after (**Post-1**; middle part of October 2018 to last part of November 2018), and three months after (**Post-2**; last part of January 2019 to middle part of March, 2019) the education, a *questionnaire regarding knowledge of early essential milk expression care among preterm mothers* (**questionnaire A**) was distributed and collected. The participants were requested to write down their characteristics (i.e., age, presence or absence of midwifery qualification, experience in the perinatal area, with or without an IBCLC certification) at Pre-1. The period of education was from the middle part of October 2018 to the last part of November 2018. The participants answered the questionnaire just before and after the implementation of the education program on the day they took part in. The participants attending the education program also answered a *questionnaire to evaluate the education program* (**questionnaire B**) at Post-2. The validity of the contents of the questionnaires was examined by several nursing research experts.

### 2.5 Measurement tool

Questionnaire A was developed in accordance with the contents of the education program. The questionnaire had 20 items as follows: Complex psychological state (Q1), Emotional support (Q2), Verification of the motivation and intent (Q3), Benefits of breastfeeding for mothers (Q4), Benefits of breast milk for infants (Q5), Significance of milk expression (Q6), Milk volume on the fourth day after birth (Q7), Milk volume at around the second week after birth (Q8), Lactogenesis stage 1 (Q9), Lactogenesis stage 2 (Q10), Lactogenesis stage 3 (Q11), Autocrine control (Q12), Blood levels of prolactin after birth (Q13), Time between birth and the initiation of milk expression (Q14), Early initiation of milk expression (Q15), Frequent milk expression (Q16), Duration of pumping (Q17), Combined use of hand expression and electric pumping (Q18), Selection of expressing method (Q19), and Consideration for breastfeeding inability (Q20). When the participants answered each item, they chose one from “*Correct*”, “*Incorrect*”, and “*Unclear*” [27]. If the answer to a question was correct, one point was given. “*Unclear*” answers and wrong answers were regarded as scoreless.

Questionnaire B was created on the basis of a previous study [30] to evaluate the acceptability (Q1: Taking an interest in care, Q2: Motivation to care, Q3: Understanding care, and Q4: Confidence in caring), demand (Q5: Necessity as nurses and midwives' skills and Q6: Necessity as practice in maternity wards), and practicality (Q7: Useful knowledge, Q8: Opportunity to reflect on care, Q9: Adopting standards of care, and Q10: Care improvement) of the program to the participants. Acceptability, demand, and practicality referred to the positive emotions to the program, necessity of the program, and utility value and usability of the program, respectively. The participants scored the 10 items on a five-point Likert scale (1 = *Strongly disagree*, 2 = *Disagree*, 3 = *Neither*, 4 = *Agree*, 5 = *Strongly agree*), and described the reason(s) for their answer in free text [27]. The lowest score was 10 points and the highest score was 50 points.

### 2.6 Data analysis

#### 2.6.1 Nurse-midwives' knowledge

McNemar’s tests were carried out between the two correct answer rates at Pre-1 and Pre-2, Pre-2 and Post-1, Pre-2 and Post-2, and Post-1 and Post-2. The Bonferroni method was used to adjust for multiple comparisons on time-series data. As the tests were conducted four times using time-series data for each knowledge item, a p-value of <0.0125 was considered to indicate a statistically significant difference. IBM SPSS Statistics version 24.0; Base and Advanced Statistics (IBM Japan, Tokyo, Japan) was used for the statistical analysis.

#### 2.6.2 Nurse-midwives' comments for improvement of the education program

In our previous study, we evaluated the acceptability, demand, and practicality of the education program from the nurse-midwives' perspective. In the present study, the main illustrative quotes in the free text section that can contribute to the improvement of the education program were extracted and reported.

### 2.7 Ethical considerations

The Research Ethics Committee of St. Luke’s International University, Tokyo, Japan approved this study (Approval number: 17-A103). All the participants were provided an oral and written explanation of the study, and they agreed to participate in writing. This research was registered in the University hospital Medical Information Network Clinical Trials Registry (Identification number: UMIN000031966).

## 3 Results

### 3.1 Participants

The lead researcher (RT) explained the present study to 44 nurses and nurse-midwives, of whom 41 agreed to participate. Of these 41 participants, five did not participate in the education program during the education period. The reasons for their non-participation were night shift full-time (n = 2), maternity leave (n = 1), sick leave (n = 1), and poor health condition (n = 1). The final number of nurse-midwives who participated in the present study was 36. Their average age was 31.8 years (range: 22–59, SD = 8.7). Their average years of experience in the perinatal area was 6.5 years (range: 0–37, SD = 8.6). Three IBCLCs were included in the final participants.

### 3.2 Nurse-midwives' knowledge

The results of McNemar’s tests on each item regarding the changes in the correct answer rates are shown in Table 1.

**Table 1:** Correct answer rates in each knowledge item between two time points.

						(n = 36)
Item	Correct answer rate (%)			p-value		
	Pre-2	Post-1	Post-2	Pre-2 & Post-1	Pre-2 & Post-2	Post-1 & Post-2
Q1 (Complex psychological state)	100.0	100.0	100.0			
Q2 (Emotional support)	100.0	100.0	100.0			
Q3 (Verification of the motivation and intent)	0.0	30.6	5.6	.001∗	.500	.004∗
Q4 (Benefits of breastfeeding for mothers)	63.9	100.0	97.2	<.001∗	<.001∗	1.000
Q5 (Benefits of breast milk for infants)	69.4	97.2	94.4	.002∗	.004∗	1.000
Q6 (Significance of milk expression)	94.4	100.0	97.2	.500	1.000	1.000
Q7 (Milk volume on the fourth day after birth)	19.4	100.0	72.2	<.001∗	<.001∗	.002∗
Q8 (Milk volume at around the second week after birth)	33.3	86.1	50.0	<.001∗	.180	.001∗
Q9 (Lactogenesis stage 1)	86.1	94.4	83.3	.453	1.000	.289
Q10 (Lactogenesis stage 2)	19.4	36.1	30.6	.109	.344	.791
Q11 (Lactogenesis stage 3)	50.0	86.1	52.8	.001∗	1.000	.004∗
Q12 (Autocrine control)	36.1	86.1	66.7	<.001∗	.013	.039
Q13 (Blood levels of prolactin after birth)	97.2	100.0	100.0	1.000	1.000	
Q14 (Time between birth and the initiation of milk expression)	30.6	72.2	55.6	<.001∗	.012∗	.109
Q15 (Early initiation of milk expression)	75.0	100.0	100.0	.004∗	.004∗	
Q16 (Frequent milk expression)	25.0	69.4	30.6	<.001∗	.754	.001∗
Q17 (Duration of pumping)	33.3	100.0	69.4	<.001∗	.001∗	.001∗
Q18 (Combined use of hand expression and electric pumping)	80.6	86.1	80.6	.754	1.000	.727
Q19 (Selection of expressing method)	58.3	77.8	61.1	.039	1.000	.210
Q20 (Consideration for breastfeeding inability)	80.6	97.2	91.7	.031	.289	.625

*Note.* ∗p < .0125.

Between the two time points Pre-1 and Pre-2, there were no significant differences in the correct answer rates for each of all the knowledge items.

The 11 knowledge items in which the correct answer rates of Post-1 were significantly higher than those of Pre-2 were as follows: Q3, Q4, Q5, Q7, Q8, Q11, Q12, Q14, Q15, Q16, and Q17.

Moreover, of these 11 knowledge items, the six knowledge items in which the correct answer rates of Post-2 were significantly higher than those of Pre-2 were as follows: Q4, Q5, Q7, Q14, Q15, and Q17. On the other hand, there were no significant differences in the correct answer rates between Pre-2 and Post-2 in the following five items: Q3, Q8, Q11, Q12, and Q16.

In the comparison between Post-1 and Post-2, the correct answer rates at Post-2 were significantly lower in the following six items: Q3, Q7, Q8, Q11, Q16, and Q17.

For the following nine items, there were no changes in the correct answer rates between Pre-2 and Post-1: Q1, Q2, Q6, Q9, Q10, Q13, Q18, Q19, and Q20.

Figure 1-1 shows the changes in the correct answer rates in the knowledge items in which the change in the correct answer rates was significant between Pre-2 and Post-1. Of the 11 items showing significant differences in the correct answer rates, the correct answer rate was low in only Verification of the motivation and intent (Q3) (range: 0.0–30.6).

**Figure 1-1:**
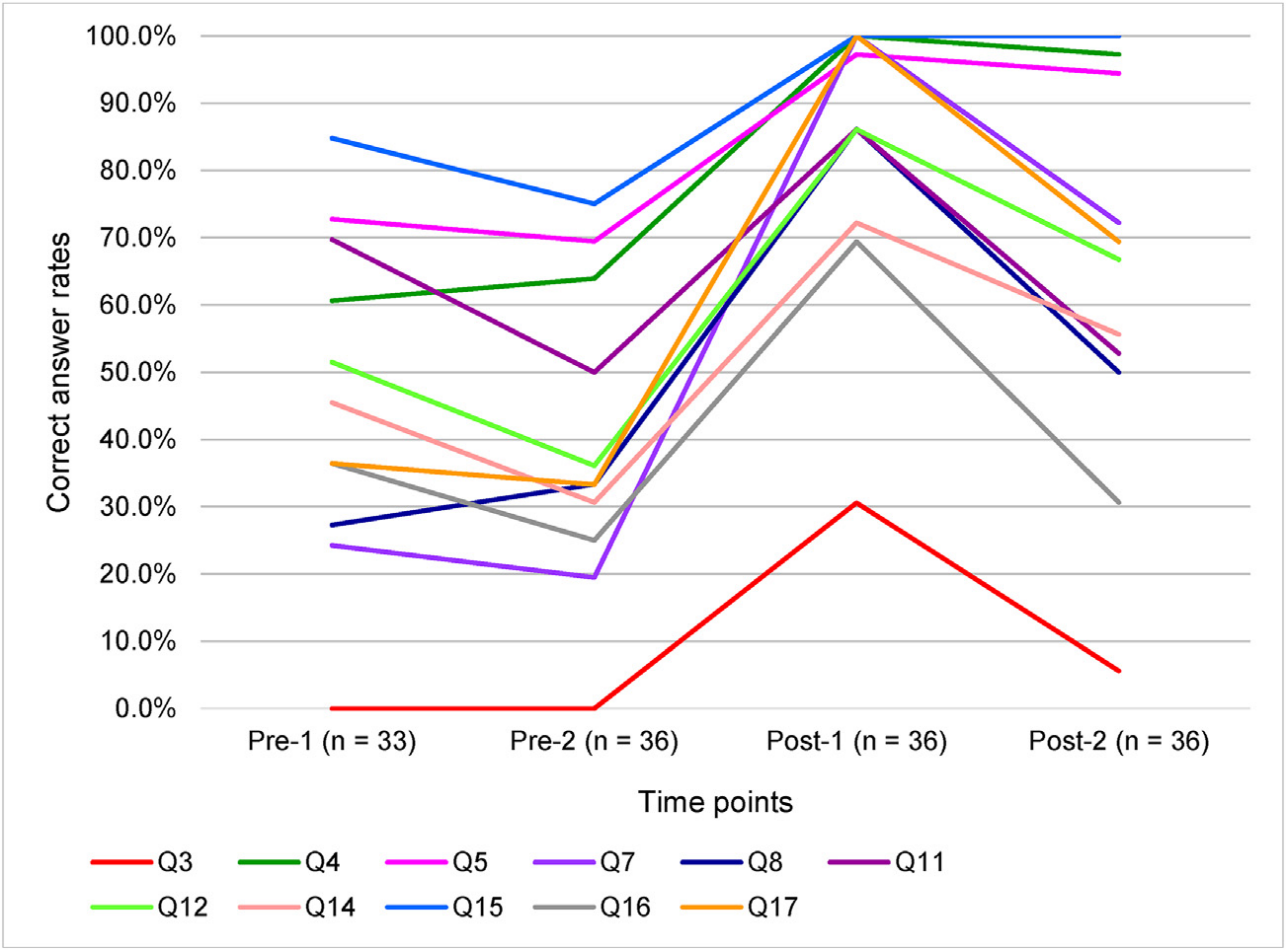
Changes in correct answer rates over time for significant knowledge items.

Figures 1-2 shows the changes in the correct answer rates in the knowledge items in which the change in the correct answer rates was not significant between Pre-2 and Post-1. Of the nine items showing no significant differences in the correct answer rates, in Complex psychological state (Q1), Emotional support (Q2), Significance of milk expression (Q6), Lactogenesis stage 1 (Q9), Blood levels of prolactin after birth (Q13), Combined use of hand expression and electric pumping (Q18), and Consideration for breastfeeding inability (Q20), the correct answer rates were already 80% or more in Pre-2. As to Lactogenesis stage 2 (Q10), the correct answer rates were from 19.4% to 39.4% through the four time-points, and that of Pre-1 was the highest. As to Selection of expressing method (Q19), the correct answer rates were from 58.3% to 77.8 % through the four time-points.

**Figure 1-2:**
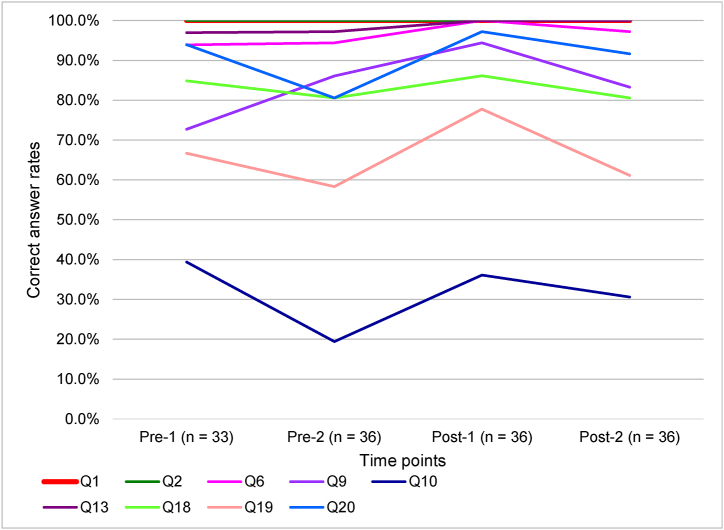
Changes in correct answer rates over time for not significant knowledge items.

### 3.3 Evaluation of the education program

One of the reasons for becoming interested in milk expression care (Q1) was expressed in this response:

*“This was a good opportunity to learn about preterm mothers because there were few lectures on it in my school days. (33)”*

There was a description indicating the misunderstanding of a nurse-midwife about the effective frequency of breast milk expression (Q3) as follows:

*“I had thought eight times of breast milk expression a day were required to maintain prolactin levels, but this time I understood six times a day were also ok. (5)”*

The negative reasons for acquiring confidence in providing milk expression care (Q4) were expressed as follows:

*“I have forgotten some parts over time because there were few opportunities to provide care and to review what I had learned in the lecture. (30)”*

There was the proposal (Q8) as follows:

*“Repetitive learning is necessary in order not to forget. (4)”*

There was a proposal (Q10) for the educational program as follows:

*“It may be better to [include in the] lecture a demonstration of the massage method. (6)”*

## 4 Discussion

In the present study, we *assessed* the effects of our educational program on *nurse-midwives'* *knowledge of breast milk expression care for preterm mothers* in terms of changes in the correct answer rates for 20 specific knowledge items before and after implementation of the education program. We also *identified* program-related issues based on the nurse-midwives' comments and *discussed* what needs to be improved in the education program.

As a result of the present study, the knowledge items improved by the education program which showed significant differences in the correct answer rates between just before and just after the education program were as follows: Verification of the motivation and intent (Q3), Benefits of breastfeeding for mothers and infants (Q4), Milk volume on the fourth day after birth (Q7), Milk volume at around the second week after birth (Q8), Lactogenesis stage 3 (Q11), Autocrine control (Q12), Time between birth and the initiation of milk expression (Q14), Early initiation of milk expression (Q15), frequent milk expression (Q16), and Duration of pumping (Q17). Therefore, this education program appeared to be effective in helping nurse-midwives acquire these knowledge items.

On the other hand, nine knowledge items were not improved by the education program which showed no significant differences in the correct answer rates between just before and just after the education program. As for the underlying reasons, of the nine knowledge items, the nurse-midwives had already most likely acquired knowledge for seven items as the correct answer rates were already 80% or more just before the education program as shown in Figure 1-2: Q1 (Complex psychological state), Q2 (Emotional support), Q6 (Significance of milk expression), Q9 (Lactogenesis stage 1), Q13 (Blood levels of prolactin after birth), Q18 (Combined use of hand expression and electric pumping), and Q20 (Consideration for breastfeeding inability). In contrast, of the nine knowledge items, the situation was different for the following two items: Q10 (Lactogenesis stage 2) and Q19 (Selection of expressing method). Regarding lactogenesis stage 2, many nurse-midwives could not understand when the mothers' milk production increases and is established. As the lactogenesis stage was explained using only letters in the lecture, it might have been better if it were explained using graphs and figures for visual understanding. As regards selection of expressing method, it was difficult to tell the nurse-midwives the importance of the selection of a comfortable method for mothers. Because a breast pump was not always effective for milk production compared with hand expression [29], it was necessary to emphasize respect for the mothers' comfort, and guidance on using both methods properly.

Regarding Q3 (Verification of the motivation and intent), although the correct answer rate increased significantly between just before and just after the education program brought about by the education program, the rate was much lower than those for the other items as shown in Figure 1-1. This suggests that understanding the content of verification of the motivation and intent may have been difficult for the nurse-midwives. This item asked about the importance of the provision of information on breastfeeding based on the mothers' motivation and intent. As mothers may hesitate to breastfeed depending on the condition of their infants, nurses should provide information according to the mothers' motivation and intent, rather than unilaterally communicating the benefits of breastfeeding to them [28]. It appeared that many participants could not understand this hesitation of mothers to breastfeed. Therefore, it may be necessary to carefully explain the context of information provision and confirmation of the mothers' motivation and intent.

Regarding the previous nurse-midwives' evaluation of the education program, most of them gave positive responses [27]. The comments of the participating nurse-midwives that would be helpful for improving the education program cannot be overlooked. For example, it was suggested that this education program is necessary for the in-service education of nurse-midwives as it was mentioned by a nurse-midwife that she lacked the opportunity to learn about breastfeeding when she was still a student. This can be inferred from the correct answer rates of each knowledge item just before the education program as shown in Table 1. In half of all the knowledge items, the correct answer rates just before the education program were <60%. Additionally, another nurse-midwife requested a demonstration of the breast massage method in this study. Sadovnikova et al. [31] reported that practical learning with a fidelity breast simulator was ideal for most healthcare professionals because they were not always able to experience various breastfeeding care when they were students. Therefore, this education program can be further improved by including practical applications such as simulations and demonstrations. We provided the participants the URL address of the teaching material on hand expression of breast milk created by Morton [32]. In addition, we should have planned for the participants to practice while watching this teaching material in the education program.

One nurse-midwife stated that she would forget some of her knowledge because of the lack of opportunities to review the lectures and practice care. Another nurse-midwife mentioned the need for repetitive learning to remember her knowledge. As a proof of this, the changes produced by the education program disappeared three months after the education program for the following items in which the change was obtained just after the education program: Verification of the motivation and intent, Milk volume at around the second week after birth, Lactogenesis stage 3, Autocrine control, and Frequent milk expression. Bowling [33] showed improvement in the retention of knowledge among the nursing staff when they repeatedly received text messages on their mobile phones after a lecture on patient fall prevention. Consequently, it is necessary to develop tools that allow nurse-midwives to continuously access knowledge about milk expression. In addition, it is necessary to consider education methods as an increase in knowledge was previously shown two weeks after receiving the education compared with the knowledge immediately after receiving the education in team-based learning about postpartum haemorrhage [34].

In this education program, one nurse-midwife misunderstood the effective frequency of breast milk expression. With respect to the frequency of milk expression, several criteria were obtained in a literature review article by Tanaka et al. [29], namely, five times or more milk expressions per day [19], 6.25 times or more per day [22], and seven times or more per day [23]. As more frequent milk expression stimulates hormone secretion for milk production in the early postpartum period, seven times or more per day was set as an appropriate frequency in this education program. However, because several lines of evidence in the process of deriving an appropriate number of milk expression were introduced in the lecture, this may have resulted in the misunderstanding and confusion of the nurse-midwife. Isaacs and Oates [35] stated that to provide a good lecture, the content needs to be clear and concise, and the slides should not be overloaded with information. Thus, education materials need to be simplified to emphasize the appropriate number of milk expression. It should also be emphasized that the number of milk expression presented in the lecture was a minimum standard and this did not mean that the number of milk expression must be reduced accordingly.

The present study is an exploratory secondary analysis of data obtained from our educational program in our previous study. It is not a study that was conducted after calculating the sample size to explain the difference in the nurse-midwives' correct answer rates for each knowledge item in the educational program. Therefore, the number of nurse-midwives (i.e., 36) was small. In our previous study, the three months time period before and after the educational program was not strictly controlled for each participant, thus the period of three months generally meant ‘about three months’.

## 5 Conclusions

The unacquired knowledge of milk expression care for preterm mothers by the nurse-midwives was effectively supplemented by the education program. Additionally, the knowledge items with low correct answer rates before the implementation of the education program must be strengthened in the in-service education of nurse-midwives. Approximately half of the knowledge items acquired by the nurse-midwives was retained even at three months after the education program.

Based on the comments of the participating nurse-midwives, the issues that can be considered for improvement of the educational program include *practical training*, *knowledge retention*, and *misunderstanding of knowledge*. These issues can be addressed by the incorporation of *practical* applications such as simulations and demonstrations, the development of tools that allow nurse-midwives to *continuously access knowledge*, and the provision of *simple and clear explanation* about knowledge of milk expression care. These improvements are needed to promote and further increase the utilization of this education program in the future.

## Declarations

### Author contribution statement

Rie Tanaka: Conceived and designed the experiments; Performed the experiments; Analyzed and interpreted the data; Wrote the paper.

Shigeko Horiuchi: Conceived and designed the experiments; Analyzed and interpreted the data; Wrote the paper.

### Funding statement

This work was supported by the individual research allowance from Teikyo University.

### Data availability statement

Data will be made available on request.

### Declaration of interest’s statement

The authors declare no conflict of interest.

### Additional information

No additional information is available for this paper.
